# Self-medication in times of illness: a multi-center cross-sectional study among medical students in Egypt

**DOI:** 10.1186/s12913-026-14296-6

**Published:** 2026-03-23

**Authors:** Yusof Mohamed Omar, Mohamed Ashraf Elsaadany, Mahmoud Hekal, Amr Khaled Abdelaal, Mohamed Hossam Ghazy, Huda Wagih, Mey E. Bastawi, Mazen Essam, Abdel-Hady El-Gilany

**Affiliations:** 1 Faculty of Medicine, Mansoura University, Mansoura, Egypt; 2 Faculty of Medicine, Cairo University, Cairo, Egypt; 3 Faculty of Medicine, Fayoum University, Fayoum, Egypt; 4 Faculty of Medicine, Sohag University, Sohag, Egypt; 5 Faculty of Medicine, Kafr El-Sheikh University, Kafr El-Sheikh, Egypt; 6 Faculty of Medicine, Qena University, Qena, Egypt; 7 Faculty of Medicine, Minia University, Minia, Egypt; 8 Public Health and Community Medicine Department, Faculty of Medicine, Mansoura University, Mansoura, Egypt

**Keywords:** Self-medication, Medication misuse, Medical students, Egypt

## Abstract

**Background:**

Medical students frequently experience health problems but often resort to self-medication instead of consulting a doctor. This practice may be influenced by various factors and is associated with serious health risks. This study aimed to assess the prevalence, predictors, and contributing factors for self-medication during illness among medical students in Egypt.

**Methods:**

A cross-sectional study was conducted among 1,128 medical students from seven public universities across three regions of Egypt. Data on self-medication practices, self-reported reasons, and associated factors were collected using an online survey distributed through university-affiliated channels. Binary logistic regression analysis was used to identify factors associated with self-medication for illness.

**Results:**

Self-medication for illness during the past year was reported by 66.6% of participants. In univariate analysis, significant predictors included older age (Crude Odds Ratio (COR) = 1.55, 95% CI: 1.21, 1.99), advancing academic year (COR range: 1.69–2.60 vs. first-year students), having a part-time job (COR = 1.68, 95% CI: 1.04, 2.73), and having a chronic illness (COR = 1.75, 95% CI: 1.11, 2.76). Self-medication was also associated with confidence in self-diagnosis (COR = 1.66, 95% CI: 1.28, 2.16), barriers to healthcare access (COR = 1.55, 95% CI: 1.21, 2.00), discrimination in healthcare settings (COR = 1.40, 95% CI: 1.01, 1.95), and delaying healthcare-seeking (COR = 1.77, 95% CI: 1.32, 2.38). In multivariable analysis, significant predictors included advancing academic year (Adjusted Odds Ratio (AOR) = 1.71, 2.26, 95% CI: 1.14, 4.03), confidence in self-diagnosis (AOR = 1.63, 95% CI: 1.25, 2.13), delaying healthcare-seeking (AOR = 1.54, 95% CI: 1.13, 2.10), and experiencing barriers to healthcare access (AOR = 1.31, 95% CI: 1.00, 1.72). The most common self-reported reasons for self-medication was lack of time (43.4%) and convenience (42.5%).

**Conclusion:**

A substantial proportion of Egyptian medical students rely on self-medication during illness, influenced by academic progression, confidence in self-diagnosis, and healthcare access barriers. Addressing these factors is crucial to promoting safer healthcare-seeking behaviors among future physicians.

**Clinical trial registration:**

Not applicable.

**Supplementary Information:**

The online version contains supplementary material available at 10.1186/s12913-026-14296-6.

## Introduction

Self-medication, defined as the use of drugs or therapeutics without professional medical guidance, is a globally widespread practice. Self-medication encompasses the use of over-the-counter medications, traditional remedies, or previously prescribed drugs for recurring symptoms, often based on personal judgment or advice from non-medical sources [1]. While self-medication can offer benefits, such as timely treatment and reduced pressure on healthcare systems [2], its risks—including adverse drug reactions [3], masking of diagnoses [4], and antimicrobial resistance [5]—make it a significant public health concern.

Self-medication poses a significant challenge in developing countries, where its prevalence is driven by a complex interplay of socioeconomic disparities, deeply ingrained cultural practices, and systemic healthcare limitations [6]. In many low- and middle-income countries such as Egypt, the easy availability of drugs without prescriptions combined with the high cost or inaccessibility of healthcare services make self-medication an attractive alternative [7, 8]. These factors lead many time-constrained individuals, particularly students, to rely on self-medication as a convenient and cost-effective option.

Among medical students, self-medication often begins during undergraduate training and is driven by their self-presumed sufficient medical knowledge [9]. Academic pressures and demanding schedules further exacerbate the issue, leaving little time to seek professional care [10]. Alarmingly, studies report that the prevalence of self-medication among medical students ranges from 12% to as high as 99%, with medical students being twice as likely to self-medicate compared to their non-medical peers [11]. This practice is not without risks, as self-medication among students can lead to adverse drug reactions and dependency on certain medications [12]. When used for self-management of illnesses, self-medication may mask symptoms, delaying diagnosis and worsening underlying conditions [4, 12].

The implications of self-medication among medical students extend beyond individual health, particularly when this behavior persists unchecked. Research suggests that physicians who develop self-medication habits during training are more likely to tolerate similar behavior in their patients, potentially undermining professionalism and eroding public trust in the medical profession [13, 14]. Additionally, self-medication can negatively affect physicians’ well-being, leading to poor health outcomes that may compromise patient care [4, 12]. Studies have also linked self-medication to later substance misuse, particularly in high-stress medical specialties, highlighting the broader risks posed by unchecked self-medication within the medical community [15].

In Egypt, self-medication is widely prevalent, even among the general population, reaching rates of nearly 62.8% [7]. However, studies on medical students have been limited and are typically restricted to single-center investigations and certain medications such as antibiotics and antifungals [9, 16], and there have yet to be any studies assessing this issue on a national level. This leaves significant gaps in understanding of the various factors associated with self-medication within this group.

Given the critical role of medical students as future prescribers, it is essential to examine their self-medication behaviors in greater depth. This study aims to fill this gap by assessing the prevalence, predictors, and reasons for self-medication among medical students in Egypt. By identifying key predictors and underlying reasons, the results may guide policy recommendations to ensure safer prescribing behaviors in their future medical practice.

## Methods

### Study design and study period

A correlational cross-sectional study was conducted between January and February 2025. This study adhered to the STROBE guidelines for reporting observational research [17].

### Outcome measure

The primary outcome was the prevalence of self-medication among medical students, measured by the question, “Have you self-medicated for health problems during the last year?” [18]. This question was selected to capture all instances of self-medication for health issues, as the potential risks of adverse effects remain significant, regardless of frequency or timing, when managing illnesses.

### Sample size

The sample size was calculated using OpenEpi software, with a 72.4% prevalence of self-medication reported in a previous study among medical students at Mansoura University [19], a precision of 5%, and a 97% confidence level. A design effect of 3 was applied to account for clustering across multiple regions and institutions, which is higher than that typically used in single-center studies but appropriate for geographically heterogeneous samples recruited using convenience sampling.

Using conventional parameters (95% confidence level), the required sample size would have been approximately 921 students after accounting for clustering. However, we adopted a higher confidence level (97%) to obtain more conservative estimates and enhance precision in a national, multi-regional sample, resulting in a final required sample size of 1128 students. A non-response rate was not added, as participants were recruited through convenience sampling without a predefined sampling frame.

### Sampling and data collection approach

A convenience sampling approach was used due to practical constraints, as there was no access to the contact information of all medical students for simple random sampling. To minimize bias, participants were recruited from seven public medical schools across three regions of Egypt: Urban Governorates (Cairo University), Lower Egypt (Mansoura and Kafr El-Sheikh Universities), and Upper Egypt (Al-Minya, Qena, Al-Fayoum, and Sohag Universities). No medical schools from frontier governorates were included, as they are recently founded and none offer a full five-year academic program in this region as of the time of the study. The sample size was distributed proportionally between different universities based on the number of registered students. The online questionnaire was distributed through Google Forms via official social channels and Telegram groups, ensuring accessibility. Participants provided informed electronic consent and completed the questionnaire anonymously at their convenience.

### Study tool

The self-administered English questionnaire used in this study was developed based on a comprehensive literature review of factors associated with self-medication behaviors and questionnaires from previous studies [19, 20]. Since the questionnaire did not include scales or constructs that required psychometric evaluation, formal validity and reliability testing was not conducted. However, to ensure its clarity and relevance, it was reviewed by two public health experts and one of the authors, a medical student, who verified its suitability for the participants, and no pilot study was conducted. The questionnaire is provided as a supplementary file (Supplementary File 1).

The first section gathered sociodemographic data potentially associated with self-medication, including sex, age, academic year, residence, family income, parental involvement in healthcare, and the presence of chronic diseases.

The second section evaluated the prevalence and reasons for self-medication for health problems among participants [18]. Those who reported self-medicating were asked to identify the primary reason, with options of: cost, convenience, time constraints, and fear of judgment when seeking healthcare.

The third section addressed healthcare access factors that may influence self-medication decisions. It included questions about students’ self-reported experiences and perceptions related to seeking medical care, exploring delays in treatment, barriers to healthcare services, confidence in self-diagnosis, and experiences of discomfort or discrimination when accessing healthcare.

### Statistical analysis

Statistical analysis was conducted using Jamovi Software for statistical analysis. The online questionnaire was configured to require responses to all items before submission. As a result, the dataset contained no missing values for variables included in the analysis. Categorical variables were summarized as frequencies and percentages. Binary logistic regression was used as the outcome variable (self-medication) was dichotomous (yes/no). Initially, univariate binary logistic regression was performed to assess the association between each independent variable and self-medication. Variables with a p-value less than 0.05 in the univariate analysis were included in the multivariable binary logistic regression model to identify independent predictors of self-medication, adjusting for potential confounders. The enter method was used to include all selected variables simultaneously in the final model. Crude odds ratios (COR), adjusted odds ratios (AOR), and their respective 95% confidence intervals (CI) were reported. Multicollinearity among independent variables in the multivariable regression model was assessed using variance inflation factors (VIFs), all of which were below 2, indicating no significant multicollinearity. A p-value less than 0.05 was considered statistically significant.

## Results

The sociodemographic characteristics of the study participants are presented in Table 1. The study included 1,128 medical students, of whom 50.8% were 21 years or older and 53.5% were female. The majority of students lived in urban areas (74.5%) with their families (62.8%), and most (93.9%) reported sufficient family income. Additionally, 25.8% had a parent in healthcare. Regarding healthcare access barriers, 79.4% reported delaying seeking professional healthcare, 43.4% reported encountering barriers to healthcare access, 19% experienced discrimination, and 68.3% expressed confidence in self-diagnosis (Table 1).

**Table 1:** Sociodemographic characteristics and univariable binary logistic predictors of self-medication among medical students in Egypt (*N* = 1128)

Variables		Total *n* (%)^1^	Self-medication *n* (%)^2^	Univariate regression	
				COR (95% CI)^3^	*P*
Overall		1128 (100)	751 (66.6%)		
Age	Less than 21	555 (49.2%)	342 (61.6%)	1 (r)	
	21 or above	573 (50.8%)	409 (71.4%)	1.55 (1.21–1.99)	**0.001**
Sex	Female	604 (53.5%)	390 (64.6%)	1 (r)	
	Male	524 (46.5%)	361 (68.9%)	1.22 (0.95–1.56)	0.125
Academic year	1st year	189 (16.8%)	98 (51.9%)	1 (r)	
	2nd year	231 (20.5%)	149 (64.5%)	1.69 (1.14–2.5)	**< 0.009**
	3rd year	291 (25.7%)	201 (69.1%)	2.07 (1.42–3.03)	**< 0.001**
	4th year	281 (24.9%)	207 (73.7%)	2.6 (1.76–3.84)	**< 0.001**
	5th year	136 (12.1%)	96 (70.6%)	2.23 (1.4–3.55)	**< 0.001**
Residence	Urban	840 (74.5%)	569 (67.7%)	1 (r)	
	Rural	288 (25.5%)	182 (63.2%)	0.818 (0.618–1.08)	0.158
Living situation	With family	708 (62.8%)	464 (65.5%)	1 (r)	
	External residence	420 (37.2%)	287 (68.3%)	1.13 (0.877–1.47)	0.336
University location	Urban Governorates	407 (36.1%)	275 (67.6%)	1 (r)	
	Upper Egypt	341 (30.2%)	210 (61.6%)	0.77 (0.57–1.04)	0.088
	Lower Egypt	380 (33.7%)	266 (70%)	1.12 (0.83–1.51)	0.462
Family income	Sufficient	1059 (93.9%)	704 (66.5%)	1 (r)	
	Less than sufficient	69 (6.1%)	47 (68.1%)	1.08 (0.639–1.82)	0.780
Parent occupation	Non-healthcare related	837 (74.2%)	554 (66.2%)	1 (r)	
	In healthcare	291 (25.8%)	197 (67.7%)	1.07 (0.806–1.42)	0.638
Part-time job	No	1031 (91.4%)	677 (65.7%)	1 (r)	
	Yes	97 (8.6%)	74 (76.3%)	1.68 (1.04–2.73)	**0.034**
Chronic illnesses	No	1016 (90.1%)	665 (65.5%)	1 (r)	
	Yes	112 (9.9%)	86 (76.8%)	1.75 (1.11–2.76)	**0.016**
Confidence in self-diagnosis	Not confident	358 (31.7%)	210 (58.7%)	1 (r)	
	Confident	770 (68.3%)	541 (70.3%)	1.66 (1.28–2.16)	**< 0.001**
Delay seeking healthcare	No	232 (20.6%)	130 (56%)	1 (r)	
	Yes	896 (79.4%)	621 (69.3%)	1.77 (1.32–2.38)	**< 0.001**
Barriers accessing healthcare	No	638 (56.6%)	398 (62.4%)	1 (r)	
	Yes	490 (43.4%)	353 (72%)	1.55 (1.21–2.00)	**< 0.001**
Discrimination in healthcare	No	914 (81%)	596 (65.2%)	1 (r)	
	Yes	214 (19%)	155 (72.4%)	1.40 (1.01–1.95)	**0.044**

^1^ Percentages are column-wise, ^2^ Percentages are row-wise, ^3^ COR = Crude Odds Ratio, CI = Confidence Interval

As shown in Tables 1 and 751 (66.6%) of the surveyed medical students reported self-medicating when experiencing health problems during the last year. Significant predictors of self-medication identified in univariate regression included being 21 years or older (COR = 1.55, 95% CI: 1.21–1.99), advancing academic year (COR = 1.69–2.6, 95% CI: 1.14–3.84), having a part-time job (COR = 1.68, 95% CI: 1.04, 2.73), and having chronic illnesses (COR = 1.75, 95% CI: 1.11, 2.76). Additionally, self-medication was significantly associated with confidence in self-diagnosis (COR = 1.66, 95% CI: 1.28, 2.16), experiencing barriers (COR = 1.55, 95% CI: 1.21, 2.00) or discrimination (COR = 1.40, 95% CI: 1.01, 1.95) when accessing healthcare, and delaying healthcare-seeking (COR = 1.77, 95% CI: 1.32, 2.38) (Table 1).

However, on multivariable analysis, significant predictors of self-medication were advancing academic year (AOR = 1.71–2.26, 95% CI: 1.14–4.03), confidence in self-diagnosis (AOR = 1.63, 95% CI: 1.25–2.13), delaying healthcare-seeking (AOR = 1.54, 95% CI: 1.13, 2.10), and experiencing barriers accessing healthcare (AOR = 1.31, 95% CI: 1.00, 1.72) (Table 2).

**Table 2:** Significant predictors of self-medication among medical students in Egypt: Multivariable logistic regression analysis

Variables		Multivariable regression		
		**β**	**AOR** **(95% CI)**^1^	**P**
Age	Less than 21		1 (r)	
	21 or above	0.221	1.05 (0.68–1.6)	0.817
Academic year	1st year		1 (r)	
	2nd year	0.206	1.71 (1.14–2.56)	**0.009**
	3rd year	0.225	1.94 (1.25–3.01)	**0.003**
	4th year	0.296	2.26 (1.26–4.03)	**0.006**
	5th year	0.329	1.86 (0.97–3.54)	0.06
Part-time job	No		1 (r)	
	Yes	0.255	1.46 (0.88–2.4)	0.139
Chronic illnesses	No		1 (r)	
	Yes	0.241	1.53 (0.95–2.45)	0.079
Confidence in self-diagnosis	Not confident		1 (r)	
	Confident	0.137	1.63 (1.25–2.13)	**< 0.001**
Delay seeking healthcare	No		1 (r)	
	Yes	0.158	1.54 (1.13–2.10)	**0.006**
Barriers accessing healthcare	No		1 (r)	
	Yes	0.137	1.31 (1.00–1.72)	**0.047**
Discrimination in healthcare	No		1 (r)	
	Yes	0.286	1.33 (0.94–1.9)	0.105

^1^ AOR = Adjusted Odds Ratio, CI = Confidence Interval

The main reasons reported for self-medicating by the study participants were lack of time (43.4%), convenience (42.5%), cost (9.5%), and fear of social stigma (4.6%) (Data not shown in tables).

## Discussion

Self-medication, a common practice among medical students, is driven by time constraints, limited access to healthcare, and the relative ease of obtaining medications. While the issue is well-documented globally, research on self-medication behaviors and associated factors among medical students in Egypt remains limited. This study sought to address this gap by exploring the prevalence, associated factors, and reasons for self-medication in this cohort, aiming to provide insights that could inform local public health policies and contribute to the promotion of safer, evidence-based healthcare practices in future medical professionals.

In this study, two-thirds (66.6%) of the surveyed medical students reported self-medicating for health problems. Our findings are slightly higher than those of a recent study among medical students in Lower Egypt, where 59.7% reported self-medicating when feeling sick [21], yet slightly lower than the 70.1% prevalence reported among medical students in the Middle Delta [22]. Notably, our results align closely with studies conducted among medical students in India [23, 24], which reported self-medication rates of 66.2% and 67.7%, respectively. Furthermore, the prevalence observed in our study is only marginally higher than the 62.8% reported among the general Egyptian population [7], suggesting that self-medication is a widespread practice in Egypt not limited to medical students.

The variation in self-medication prevalence across studies may stem from differences in how self-medication was measured. While our study specifically examined self-medication for illness, others assessed the practice more broadly or using specific drugs [23, 24]. Additionally, in Egypt, the widespread availability of prescription medications without a prescription likely facilitates self-medication, allowing students to bypass healthcare consultations [8]. This unrestricted access raises significant public health concerns, increasing the risk of improper medication use, adverse drug reactions, and more serious health complications [4, 12]. Consistent with these concerns, our findings suggest a significant association between self-medication and delays in seeking healthcare. Such delays not only exacerbate underlying conditions but may also contribute to poorer long-term health outcomes, reinforcing the need for targeted interventions and policies to address this issue.

Nonetheless, the relative ease of medication access may not fully explain the prevalence of self-medication, as our findings also reveal a significant association between self-medication and both advancing academic year and confidence in self-diagnosis. These findings align with previous studies among medical students in Egypt [21, 25], Afghanistan [26], and Zambia [27], which similarly reported a significant link between educational stage and self-medication. However, unlike our findings, a study among medical students in Turkey found no statistically significant difference in self-medication rates across academic years [28]. Furthermore, our results also contrast those of a study among university students in Saudi Arabia [29], in which older students were significantly less likely to report self-medicating.

As medical students progress through their academic years, their increasing medical knowledge often fosters greater confidence in self-diagnosis, making them more likely to self-medicate rather than seek professional care. Among more senior students, consulting a doctor may even be perceived as a sign of weakness or a lack of competence, further reinforcing the tendency to rely on self-medication [30]. This mindset, while rooted in a desire for self-sufficiency, carries significant risks, including potential adverse effects, misdiagnosis, and delays in appropriate treatment, especially when self-medicating for health problems [12]. Encouraging students to recognize that consulting a healthcare provider is a responsible and essential aspect of patient care—rather than a reflection of inadequacy—is crucial for fostering safer healthcare practices. This is particularly important given that studies have consistently shown medical students’ tendency to delay seeking proper medical care, often citing reasons such as perceived time constraints and barriers to accessing healthcare [31, 32].

Barriers to accessing healthcare may drive students toward self-medication as a more convenient alternative. Prolonged waiting times, long queues, and perceived discrimination in healthcare settings have been cited as key reasons for this practice among medical students [27, 33], including in our study. Such barriers may discourage timely healthcare seeking and reinforce a culture of self-reliance, particularly among senior students who perceive self-diagnosis as sufficient. Given these findings, addressing self-medication among medical students requires a multifaceted approach that not only improves healthcare accessibility but also fosters a culture that encourages students to prioritize their own well-being and seek timely medical attention when needed.

From a policy and educational perspective, our findings highlight several actionable interventions within Egyptian medical schools. At the institutional level, medical schools could integrate structured training on rational drug use, self-care boundaries, and appropriate healthcare-seeking behaviors into undergraduate curricula, particularly during clinical years when confidence in self-diagnosis increases. In parallel, improving access to confidential, student-friendly healthcare services such as on-campus clinics or designated referral pathways for medical students may reduce delays in seeking care. Further, strengthening the enforcement of prescription-only medication regulations may help limit unsupervised access to medications. Together, these measures could promote safer medication practices, encourage timely professional consultation, and foster a culture that views seeking healthcare as a professional responsibility rather than a sign of weakness.

### Strengths and limitations

A key strength of this study is its multi-centric design, which enhances the generalizability of the findings. Our study also examined a wide range of factors influencing self-medication, offering a more comprehensive understanding of this behavior. By specifically assessing self-medication for health problems rather than general medication use, the study provided a clinically relevant perspective on its risks and implications.

However, some limitations should be acknowledged. The use of convenience sampling may limit the representativeness of the sample. Future studies should consider probability-based sampling methods to improve external validity. The reliance on self-reported data introduces the possibility of recall and social desirability biases. Additionally, some questionnaire items may have restricted the depth of responses. For example, participants were asked to select only one reason for self-medication, and variables such as confidence in self-diagnosis, delayed healthcare seeking, and barriers to access were measured using binary items. Dichotomizing these complex constructs may have led to misclassification and loss of information, potentially biasing effect estimates toward the null by reducing variability. Conversely, in some instances, binary categorization may have inflated associations by masking gradations in behavior or attitude. As a result, the observed associations should be interpreted as conservative approximations rather than precise estimates of effect size. Lastly, the cross-sectional design precludes causal inferences, highlighting the need for longitudinal studies to assess changes in self-medication behaviors over time and their long-term effects.

## Conclusions

This study provides a comprehensive assessment of self-medication among medical students in Egypt, revealing a high prevalence of the practice driven by academic progression, confidence in self-diagnosis, barriers to healthcare access, and delays in seeking medical care. The findings highlight the need for targeted interventions to address self-medication behaviors, including stricter regulation of prescription medications, enhanced medical curricula on rational drug use, and improved access to student healthcare services. Given that medical students are future healthcare providers, fostering responsible healthcare-seeking behaviors during their training is crucial to ensuring safer prescribing practices and mitigating the risks associated with self-medication. Future research should explore longitudinal trends and the potential long-term consequences of self-medication among medical students.

## Supplementary Information

Below is the link to the electronic supplementary material.

Supplementary Material 1

## Data Availability

The data of this study is available from the corresponding author upon reasonable request.

## Abbreviations

AOR: Adjusted Odds Ratio
CI: Confidence Interval
COR: Crude Odds Ratio
STROBE: Strengthening the Reporting of Observational Studies in Epidemiology
VIF: Variance Inflation Factor
