# Supplementary material for: Self-medication in times of illness: a multi-center cross-sectional study among medical students in Egypt
Source: BMC Health Serv Res. 2026 Mar 23;26:492. doi: 10.1186/s12913-026-14296-6 (PMC13063951; doi:10.1186/s12913-026-14296-6)
Supplement: Supplementary file 1 — Supplementary Material 1 [file 12913_2026_14296_MOESM1_ESM.docx]

# Study Questionnaire

# Informed consent:

# I voluntarily agree to participate in this research study.

# I understand that even if I agree to participate now, I can withdraw at any time or refuse to answer any question without any consequences of any kind.

# I understand that participation involves answering the following questions, which will be processed and analyzed only by the researchers on this team.

# I understand that I will not benefit directly from participating in this study.

# I understand that all information I provide for this study will be treated confidentially.

## Section 1:

**1. Age:** (in years)

**2. Sex:** - 1. Male
 - 2. Female

**3. Academic Year:** - 1. 1st year
 - 2. 2nd year
 - 3. 3rd year
 - 4. 4th year
 - 5. 5th year

**4. Residence:** - 1. Urban (City)
 - 2. Rural (Countryside)

**5. Living Situation During Academic Year:** - 1. With family
 - 2. External residence

**6. Medical School:** (Specify university name)

**7. Family Income:** - 1. Sufficient
 - 2. Less than sufficient

**8. Parent occupation:** - 1. Healthcare-related occupation
 - 2. Non-healthcare related occupation

**9. Part-Time Job:** - 1. Yes
 - 2. No

**10. Do you suffer from any chronic diseases?** - 1. Yes
 - 2. No

## Section 2:

## 1. Have you self-medicated for health problems during the last year? - 1. Yes - 2. No

[Note from authors: future researchers are encouraged to include a brief definition of "self-medication" to ensure consistent interpretation]

**2. What is the main reason you choose to self-medicate?** - 1. Cost
 - 2. Convenience
 - 3. Fear of social stigma
 - 4. Lack of time

## Section 3:

**1. How confident are you in your ability to self-diagnose health problems?** - 1. Not confident
 - 2. Confident

**2. Do you ever delay seeking healthcare?** - 1. Yes
 - 2. No

**3. Do you face any barriers accessing healthcare services?**  - 1. Yes
 - 2. No

**4. Do you feel discriminated against when seeking healthcare services?** - 1. Yes
 - 2. No
